# LIX1 regulates YAP activity and controls gastrointestinal cancer cell plasticity

**DOI:** 10.1111/jcmm.15569

**Published:** 2020-07-07

**Authors:** Amandine Guérin, Delphine Martire, Eva Trenquier, Tom Lesluyes, Sébastien Sagnol, Marine Pratlong, Elise Lefebvre, Fréderic Chibon, Pascal de Santa Barbara, Sandrine Faure

**Affiliations:** ^1^ PhyMedExp University of Montpellier, INSERM, CNRS Montpellier France; ^2^ Cancer Research Center of Toulouse University of Toulouse, INSERM, CNRS Toulouse France; ^3^ MGX Biocampus Montpellier, CNRS, INSERM University of Montpellier Montpellier France

**Keywords:** cancer, gastrointestinal, LIX1, sarcoma, signaling pathway, smooth muscle, YAP1

## Abstract

Gastrointestinal stromal tumours (GISTs), the most common mesenchymal neoplasm of the gastrointestinal tract, result from deregulated proliferation of transformed KIT‐positive interstitial cells of Cajal that share mesenchymal progenitors with smooth muscle cells. Despite the identification of selective KIT inhibitors, primary resistance and relapse remain a major concern. Moreover, most patients develop resistance partly through reactivation of KIT and its downstream signalling pathways. We previously identified the *Limb Expression 1* (*LIX1*) gene as a unique marker of digestive mesenchyme immaturity. We also demonstrated that LIX1 regulates mesenchymal progenitor proliferation and differentiation by controlling the Hippo effector YAP1, which is constitutively activated in many sarcomas. Therefore, we wanted to determine LIX1 role in GIST development. We found that LIX1 is strongly up‐regulated in GIST samples and this is associated with unfavourable prognosis. Moreover, LIX1 controls GIST cell proliferation in vitro and in vivo. Upon *LIX1* inactivation in GIST cells, YAP1/TAZ activity is reduced, KIT (the GIST signature) is down‐regulated, and cells acquire smooth muscle lineage features. Our data highlight LIX1 role in digestive mesenchyme‐derived cell‐fate decisions and identify this novel regulator as a target for drug design for GIST treatment by influencing its differentiation status.

## 1 INTRODUCTION

The digestive musculature arises from the differentiation of common mesenchymal progenitors into interstitial cells of Cajal (ICCs) and smooth muscle cells (SMCs).^1^, ^2^ Unlike many other mature cell types in the adult body, smooth mesenchyme‐derived cells do not terminally differentiate, but they can reversibly modulate their phenotype, switching between a differentiated functional quiescent state and a highly proliferative mesenchymal precursor state. This feature is often associated with high neoplastic risk.^3^ Gastrointestinal stromal tumours (GISTs) are the most common mesenchymal cancers of the gastrointestinal tract.^4^ GISTs result from deregulated proliferation of KIT‐positive cells, either ICCs or ICC/SMC mesenchymal progenitors.^1^, ^4^, ^5^, ^6^ GISTs occur predominantly in the stomach (50%‐60%) and small intestine (30%‐35%). More than 80% of GISTs harbour KIT gain‐of‐function mutations that result in the constitutive activation of the KIT receptor and downstream signalling pathways, leading to spontaneous proliferation and uncontrolled tumour growth.^7^ Low‐risk tumours can be managed by surgery alone; however, approximately 50% of GISTs relapse or metastasize after surgical resection. Imatinib mesylate, a small‐molecular tyrosine kinase inhibitor against constitutively activated KIT, is efficient in adult patients with GIST.^8^ However, resistance to imatinib mesylate is increasing and complete remission is rare, highlighting the necessity to improve our molecular understanding of GIST pathophysiology.^5^

Cell dedifferentiation is a central mechanism in the initiation of neoplastic transformation and therapeutic resistance.^9^, ^10^, ^11^ It involves loss of lineage‐specific gene expression and regression from a specialized tissue to a more primitive state of development through expression of genes that govern embryonic cell‐fate specification. Accordingly, developmental biology studies are very useful for the identification of new tumour markers and therapeutic targets.^12^ In this context, we previously performed a screen to identify genes with high expression at the earliest stages of stomach development and found that *Limb Expression 1* (*LIX1*) is a novel and unique marker of stomach mesenchymal progenitors. Moreover, we demonstrated that LIX1 stimulates the expression and activity of the Hippo effector YAP1 and that both LIX1 and YAP1 are key regulators of stomach mesenchymal progenitor development.^13^, ^14^ Although *LIX1* transcripts have been detected in human GIST cell lines,^15^ LIX1 role in GIST is unknown.

Here, we found that LIX1 is strongly up‐regulated in GIST specimens and that its expression is associated with poor prognosis. LIX1 down‐regulation reprogrammes KIT‐positive GIST cells towards the SMC lineage, thereby limiting their tumorigenic and malignant potential. Therefore, our study reveals LIX1 key role in GIST pathophysiology as a rheostat for the control of cell identity.

## 2 METHODS

### 2.1 Cell culture and reagents

The GIST‐T1 cell line was from Cosmo Bio. It was established from a metastatic human GIST sample with a heterozygous deletion of 57 bases in exon 11 of KIT.^16^ Human gastric SMCs, provided by Innoprot Innovative, were grown in Dulbecco's modified Eagle medium (DMEM) supplemented with 10% foetal bovine serum (FBS) and 1% penicillin/streptomycin. GIST‐T1 cells were resuspended in Accutase™ solution (Sigma‐Aldrich) before electroporation of different constructs using the Neon Transfection System (Life Technologies), according to the manufacturer's instructions. GIST‐T1 stable cell lines were generated by selection in 500 ng/mL puromycin. All cell lines were routinely tested for the absence of mycoplasma contamination (VenorGeM OneStep Test, BioValley). Verteporfin (Sellekchem) was added to GIST‐T1 cells at a final concentration of 2 μmol/L.

### 2.2 Human GIST tissue microarrays (TMA) and GIST data set

The SuperBiochips GIST TMA (#DAA2) was purchased from Super BioChips laboratories. It contained 40 formalin‐fixed, paraffin‐embedded (FFPE) human GIST specimens and nine matched normal gut tissue specimens. The clinicopathological features of these patients are in Table S1. The second GIST TMA (#A225, BIOCAT, GmbH) contained 37 FFPE human GIST specimens and four non‐neoplastic specimens. The clinicopathological features are in Table S2. For immunohistochemistry, TMAs were rehydrated through Histoclear (Fisher Scientific) and graded alcohol solutions, and then heated at 90°C in 0.01 mol/L citrate buffer (pH 6.0) for epitope unmasking. Spots were evaluated by three examiners blinded to the clinicopathological information. Immunoreactivity was considered positive when signal was above the background signal in the negative control. Staining intensity was scored as negative (−), intermediate (+) or high (++). A clinically annotated gene expression data set of localized, untreated GISTs (n = 60), quantified by microarray, was also used.^17^

### 2.3 *shRNAs* and DNA plasmids

The human *LIX1*‐specific short hairpin RNA (shRNA) constructs pGFP‐C‐*LIX1*‐shRNALenti (TL303518A for sh*LIX1#1*: 5′‐AGTGTTCAGGAAGCAGTAGCCTCCACCAG‐3′ and TL303518B for sh*LIX1#2*: 5′‐AGCCAGGAAAAGCAGGACAAGAACTACGGT‐3′) were obtained from Origene Technologies. The 29‐mer *Scrambled* shRNA construct pGFP‐C‐*Scr*‐shLenti (TR30021 for *Scramble*: 5′‐GCACTACCAGAGC‐TAACTCAGATAGTACT‐3′) from Origene Technologies was used as control. SMARTpool ON‐TARGETplus human *YAP1* siRNA (L‐016083‐00‐0010) and ON‐TARGETplus *WWTR1* siRNA (L‐016083‐00‐0010) were from DHARMACON‐GE.

### 2.4 Cell proliferation and wound healing assays

BrdU incorporation was performed using the Cell Proliferation ELISA, BrdU (colorimetric) (ROCHE) following the manufacturer's protocol. For wound healing experiments, 1.2 × 10^6^ cells (Scrambled, GIST‐T1‐*ShLIX1#1* and GIST‐T1‐*ShLIX1#2*) were plated in 6‐well plates to reach 100% confluence 24 hours later. Then, a linear scratch in the cell monolayer was made using a 200 µL micropipette tip. Medium and cell debris were aspirated, followed by two washes with PBS. DMEM containing 2% SVF, penicillin and streptomycin, and 1 µmol/L aphidicolin (an inhibitor of cell growth) was carefully added to each well. The wound area was monitored by video‐microscopy from 0 to 48 hours post‐wounding and measured with the ImageJ software.

### 2.5 Reverse transcription and quantitative polymerase chain reaction (RT‐qPCR)

Total RNA was extracted from cell cultures with the HighPure RNA Isolation kit (Roche). Reverse transcription was performed using the Verso cDNA synthesis kit (Thermo Scientific) and qPCR using the LightCycler technology (Roche Diagnostics). PCR primers (Table S3) were designed using the LightCycler Probe Design 2.0 software. Expression levels were determined with the LightCycler analysis software (version 3.5) relative to standard curves. Data are the mean level of gene expression relative to the expression of the reference genes *GAPDH* and *RPLPO* calculated using the 2^−ΔΔCT^ method.

### 2.6 Western blotting

Cells were resuspended in lysis buffer (20 mmol/L Tris pH8, 50 mmol/L NaCl, 1% NP40, cOmplete EDTA‐free Protease Inhibitor Cocktail [Roche]). For Western blot analysis, 10 μg of total protein lysates were boiled in SDS‐PAGE sample buffer, separated by 10% SDS‐PAGE and transferred to nitrocellulose membranes. Membranes were incubated with primary antibodies. Antibodies are listed in Table S4.

### 2.7 Cell fixation, immunofluorescence microscopy, live cell imaging, quantification

Cells were seeded on fibronectin‐coated (50 μg/mL per coverslips) coverslips, fixed with 4% paraformaldehyde in PBS containing 0.01% Triton X‐100 for 10 minutes, blocked with 1% goat serum for 1 hour before incubation with primary and secondary antibodies (Alexa 350‐, 488‐ and 555‐conjugated secondary antibodies [Life Science]) in 0.1% goat serum (Table S4). Nuclei were labelled with Hoechst (Invitrogen).

### 2.8 RNA sequencing, sequencing quality control and RNA‐Seq data analysis

Libraries from GIST‐T1‐*Scrambled*, ‐*ShLIX1#1* and ‐*ShLIX1#2* cells were constructed using the TruSeq Stranded mRNA Library Prep Kit (Illumina, ref.RS‐122‐2101) according to the manufacturer's instructions. Briefly, poly‐A RNA was purified using oligo‐d(T) magnetic beads, fragmented and reverse transcribed using random hexamers, Super Script II (Life Technologies, ref. 18064‐014) and actinomycin D. During the second strand generation step, dUTP was added instead of dTTP to prevent the use of the second strand as template during the final PCR amplification. Double‐stranded cDNA was adenylated at the 3′ end before ligation using Illumina indexed adapters. Ligated cDNA was amplified by 15 cycles of PCR and PCR products were purified using AMPure XP Beads (Beckman Coulter Genomics, ref. A63881). Libraries were validated using a Fragment Analyzer (Agilent) and quantified using the KAPA Library quantification kit (Roche, ref. KK4824). Nine libraries were pooled in equimolar amounts and sequenced on an HiSeq2500 using the single read protocol (50 nt; 1.5 lane flowcell). Image analysis and base calling were performed using the Illumina HiSeq Control Software and the Real‐Time Analysis component. Demultiplexing was performed using the Illumina conversion software (bcl2fastq 2.18). The raw data quality was assessed using FastQC from the Babraham Institute and the Illumina software SAV (Sequencing Analysis Viewer). Potential contaminants were monitored with the FastQ Screen software from the Babraham Institute.

RNA‐seq reads were aligned to the human genome (UCSC Hg38) with the splice junction mapper TopHat 2.1.1^18^ and Bowtie 2.2.9.^19^ Gene model annotations were downloaded from the UCSC database (14 January 2019). Final read alignments with more than three mismatches were discarded. Reads for each gene were counted using the union mode of HTSeq‐count 0.9.0.^20^ Before statistical analysis, genes with fewer than 15 reads (cumulating all the analysed samples) were filtered out. Counts were normalized using the Relative Log Expression (RLE) method as implemented in the Bioconductor^21^ package EdgeR 3.20.1.^22^ Differentially expressed (DE) genes were identified using three different statistical methods: DESeq 1.30.0,^23^ EdgeR and DESeq2 1.18.1.^24^ The *P*‐values for multiple testing were corrected using the Benjamini‐Hochberg FDR method.^25^ Genes with adjusted *P*‐value < .001 were classified as ‘differentially expressed’. The final lists of DE genes (2767 genes) included the genes identified by all three tests and by the two comparisons (GIST‐T1‐*ShLIX1#1* vs GIST‐T1‐*Scrambled* and GIST‐T1‐*ShLIX1#2* vs GIST‐T1‐*Scrambled*). DE genes were then divided in up‐regulated genes (n = 1453) and down‐regulated genes (n = 1314) in both the GIST‐T1‐*ShLIX1#1* vs GIST‐T1‐*Scrambled* and GIST‐T1‐*ShLIX1#2* vs GIST‐T1‐*Scrambled* comparisons. Genes with different directions of regulation in the two comparisons were removed. The functional analysis of the resulting DE genes was performed with the Gene Ontology (GO) annotations and the topGO^26^ package from Bioconductor. The IDs of the DE genes were retrieved from NCBI (24 January 2019). Overrepresented GO terms were identified using Fisher's exact test with the weight method implemented in the topGO package. As confidence threshold, a *P*‐value of .001 was used. To perform this analysis, DE genes were compared with all known genes present in the annotation. The GO categories were found in the Org.Hs.eg.db package based on the gene reporter EntrezGeneID. Plots were generated using GOplot v1.0.2 in R.

### 2.9 Chorioallantoic membrane (CAM) assay

Fertilized White Leghorn eggs from Les Bruyeres Farm (France) were incubated at 38°C in humidified incubators. After 2 days of incubation, 3 mL of albumin was removed with a sharp needle, and an approximately 10 mm opening was created at the top by carefully minimizing any contamination of the interior with shell fragments. The opening was sealed with scotch‐tape and eggs incubated for 5 days. At day 7 of embryonic development (E7), the opening was enlarged to about 20 mm to allow grafting. For each condition, 1 × 10^6^ GIST‐T1 cells were resuspended in 25 μL serum‐free medium and 25 μL Matrigel Matrix (BD Biosciences). After polymerization into a drop at 37°C for 5 minutes, cells were implanted at the top of the CAM. At day 5 post‐graft, the GIST cell grafts were excised with the surrounding CAM, fixed in 4% paraformaldehyde, embedded in paraffin, cut in 10 μm sections, and processed for histological (haematoxylin‐eosin solution) or immunohistochemistry analysis, as previously described.^6^, ^27^ Images were acquired using a Nikon‐AZ100 stereomicroscope.

### 2.10 Statistical analysis

Data were analysed with the two‐tailed or when appropriate, one‐tailed Mann‐Whitney test using the GraphPad Prism 6 software. Results were considered significant when *P* < .05 (*), *P* < .01 (**), *P* < .001 (***) or *P* < .0001 (****).

## 3 RESULTS

### 3.1 LIX1 expression is a negative prognostic factor in GIST

**FIGURE 1:**
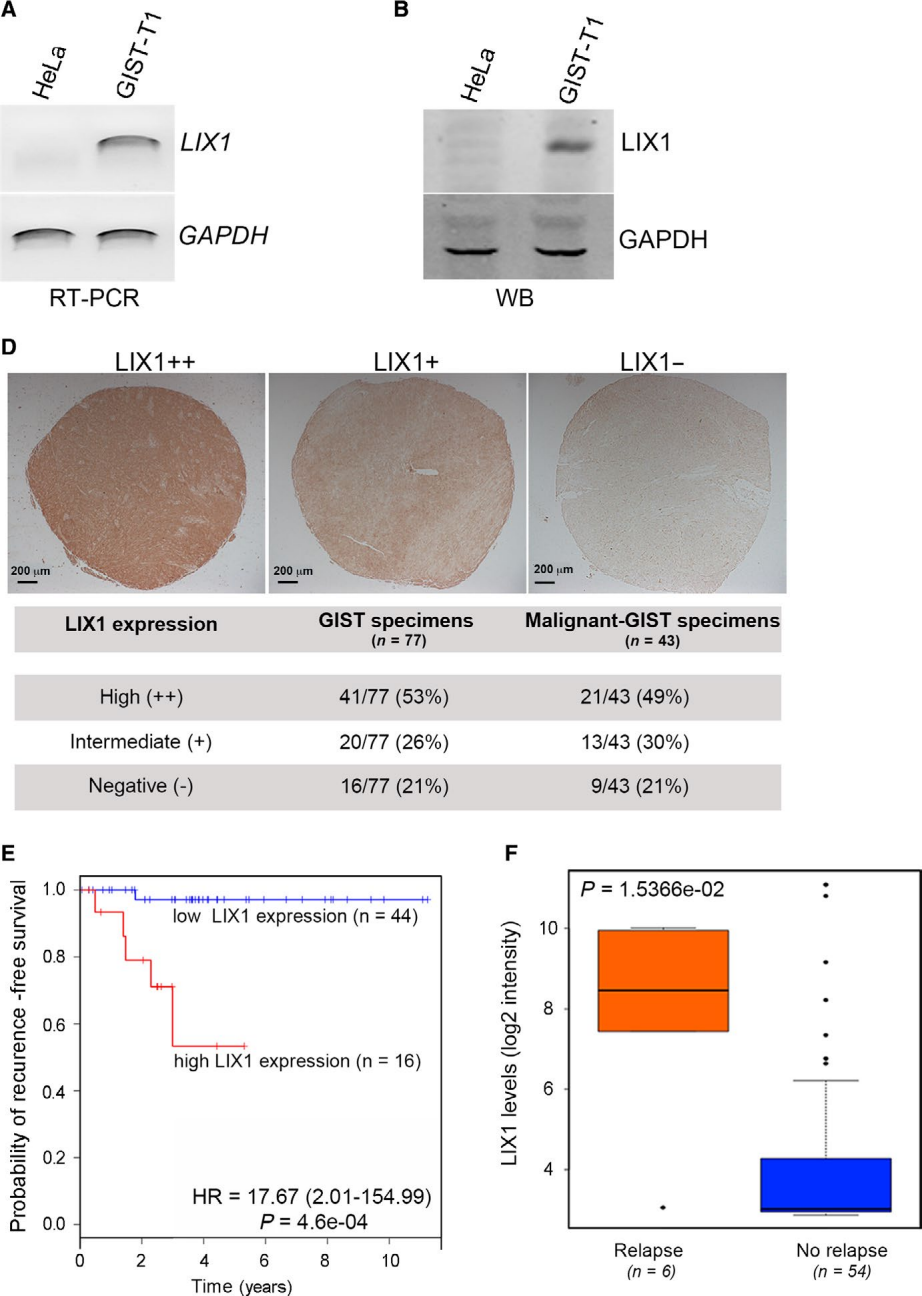
*LIX1* is expressed in GIST samples and the GIST‐T1 cell line. A, Semi‐quantitative RT‐PCR analysis of *LIX1* transcript levels in HeLa (control) and GIST‐T1 cells. Loading was verified by *GAPDH* expression. B, Western blot analysis of LIX1 protein expression in HeLa (control) and GIST‐T1 cells. GAPDH was used as loading control. C, LIX1 expression in two GIST Tissue Microarrays. Representative examples of GIST samples showing strong (LIX1++), moderate (LIX1+) and negative (LIX1−) LIX1 expression. Among the 77 GIST specimens, 43 were high‐grade GISTs. Scale bars, 200 μm. D, Kaplan–Meier curve of progression‐free survival using data from the ATGsarc microarray database for 60 patients with GIST. Patients were divided in Group 1 (low *LIX1* level) and Group 2 (high *LIX1* level). *P*‐values of the log‐rank test are indicated. n = number of patients in each group. E, Correlation between *LIX1* transcript levels and local relapse. Relapse, n = 6; No relapse n = 54. In five of the six tumours that relapsed, *LIX1* expression level was higher than the mean value

Gastrointestinal stromal tumours result from the deregulated proliferation of transformed KIT‐positive ICCs that share mesenchymal progenitors with SMCs. Neoplastic transformation requires changes in cell identity and acquisition of progenitor‐like features.^28^ As LIX1 regulates the proliferation of muscle progenitors^13^, ^29^ and cell‐fate decisions within the digestive mesenchymal lineage,^13^ it could be implicated in GIST pathogenesis. In the course of this study, another group reported that *LIX1* is expressed in two GIST cell lines.^15^ Here, analysis of LIX1 expression in an another cell line (GIST‐T1) that was derived from a metastatic human GIST with a heterozygous deletion of 57 bases in exon 11 of *KIT* ^16^ confirmed LIX1 (mRNA and protein) expression (Figure 1A,B). Furthermore, immunohistochemical analysis of LIX1 expression in two GIST TMAs showed that LIX1 was expressed in 61 (79%) of all GIST specimens (n = 77), and in 34/43 high‐grade GISTs (79%) (Figure 1C). To further understand LIX1 influence on the clinical outcome of patients with GIST, we analysed recurrence‐free survival in function of LIX1 expression in a previously described clinical data set^17^ using Kaplan‐Meier curves and the log‐rank test. This data set included 60 patients with GIST and high (n = 16) or low (n = 44) *LIX1* expression (median follow‐up of 58 months). Recurrence‐free survival was significantly reduced in the group with high *LIX1* expression (relative risk = 17.669, 95% confidence interval 2.01‐154.99, log‐rank test, *P* = .0005) (Figure 1D). *LIX1* expression was highest in patients with relapsed tumour (*P* = .015) (Figure 1E). These findings identify *LIX1* expression as a novel prognostic factor in GIST.

### 3.2 LIX1 down‐regulation decreases YAP1/TAZ and KIT levels in GIST cells

**FIGURE 2:**
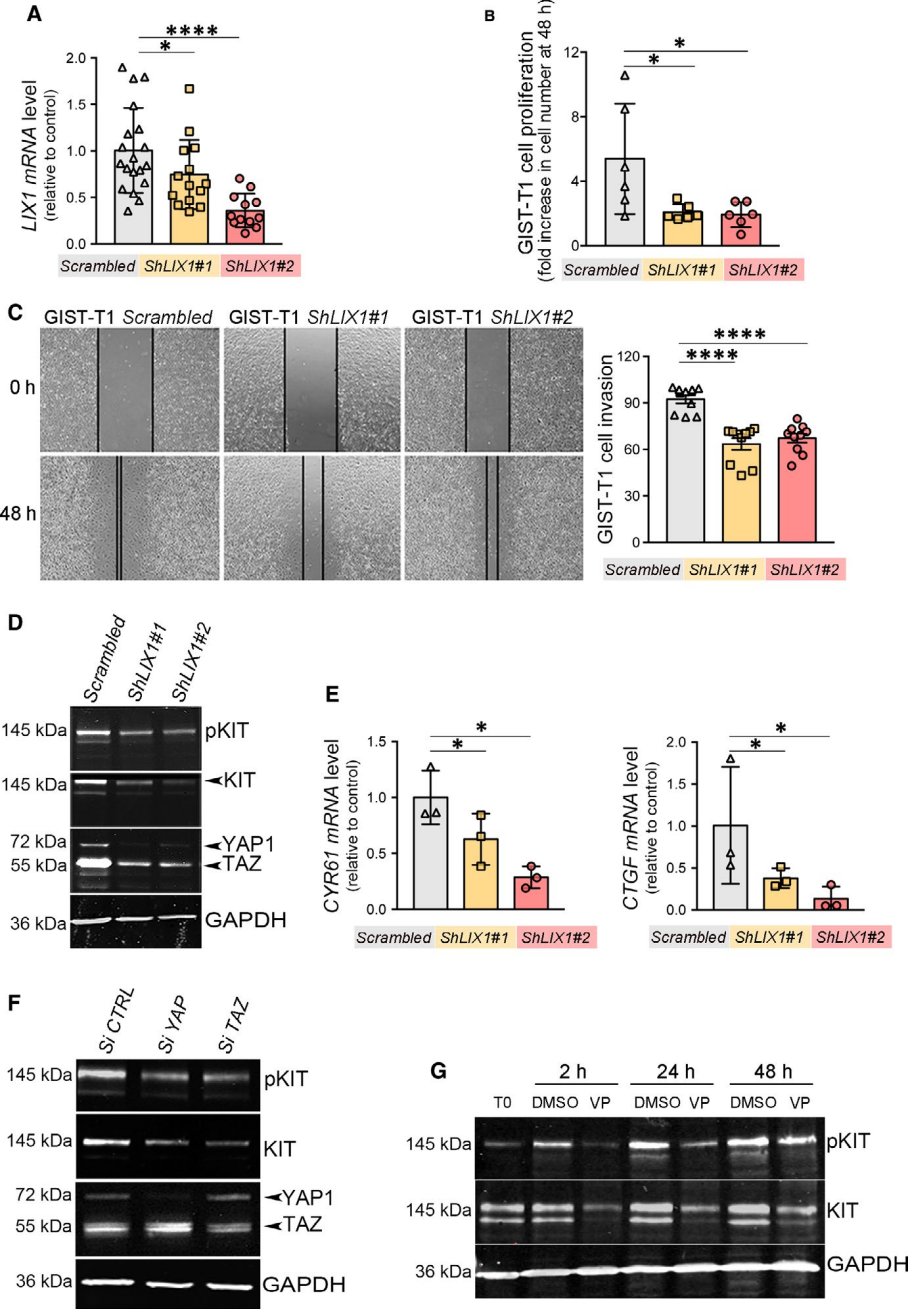
LIX1 down‐regulation reduces the proliferative and invasive capacities of GIST cells. A, RT‐qPCR analysis of *LIX1* transcript level in GIST‐T1 cells upon *LIX1* silencing. Data were normalized to the mean *GAPDH* and *RPLPO* expression. Normalized expression levels were converted into fold changes. Values are presented as the mean ± SEM of n = 20 samples of GIST‐T1‐*Scrambled*, n = 15 for GIST‐T1‐*ShLIX1#1* and n = 12 for‐*ShLIX1#2* cells. *P < .05 and ****P < .0001 (one‐tailed Mann–Whitney test). B, Cell proliferation analysis (BrdU incorporation) in GIST‐T1‐*Scrambled*, GIST‐T1‐*ShLIX1#1* and ‐*ShLIX1#2* cells. Cells were cultured for 2 d. Values are the mean ± standard derivation (SD) of n = 6 independent experiments. *P < .05 (two‐tailed Mann–Whitney tests). C, Wound healing assays of GIST‐T1‐*Scrambled*, GIST‐T1‐*ShLIX1#1* and ‐*ShLIX1#2* cells cultured for 48 h after wounding. Graph represents the percentage of wound closure at 48 h post‐scratch. ****P < .0001 (two‐tailed Mann–Whitney test). D, Representative western blot showing phosphorylated KIT (pKIT), KIT and YAP1/TAZ levels in GIST‐T1‐*Scrambled*, GIST‐T1‐*ShLIX1#1* and ‐*ShLIX1#2* cells. Equal loading was verified by GAPDH expression. E, RT‐qPCR analysis of *CYR61* and *CTGF* expression. Data were normalized to the mean *GAPDH* and *RPLPO* expression, and converted to fold changes. Values are the mean ± SEM of n = 3 samples of GIST‐T1‐*Scrambled*, n = 3 for GIST‐T1‐*ShLIX1#1*, and n = 3 for‐*ShLIX1#2* cells. *P < .05 (one‐tailed Mann–Whitney test). F, Representative western blot showing YAP1/TAZ, phosphorylated KIT (pKIT) and KIT levels in GIST‐T1‐*Scrambled*, GIST‐T1‐*SiYAP* and GIST‐T1‐*SiTAZ* cells. Equal loading was verified by GAPDH expression. G, Representative western blot showing phosphorylated KIT and KIT expression in GIST‐T1 cells untreated (T0) or incubated with 2 μmol/L verteporfin (VP) or DMSO (vehicule) for 2, 24 or 48 h. Equal loading was verified by GAPDH expression

As increased expression of LIX1 is associated with unfavourable prognosis in GISTs, we determined whether LIX1 regulates the growth of GIST cells in vitro. First, we established GIST cell lines that stably express GIST‐T1‐*Scrambled* (negative control shRNA) and shRNAs against *LIX1* (GIST‐T1‐*ShLIX1#1* and GIST‐T1‐*ShLIX1#2*). RT‐qPCR analysis confirmed *LIX1* down‐regulation in GIST‐T1‐*ShLIX1* cells (Figure 2A), particularly in cells transfected with *ShLIX1#2*. Proliferation assays (BrdU incorporation) in GIST‐T1‐*Scrambled* and GIST‐T1‐*ShLIX1* cells showed that *LIX1* silencing led to a significant reduction of cell proliferation compared with GIST‐T1‐*Scrambled* cells (Figure 2B). Then, to assess the effect of *LIX1* silencing on GIST cell migration we performed wound healing assays. Scratch wound closure was slower in GIST‐T1‐*ShLIX1#1* and GIST‐T1‐*ShLIX1#2* cells than in GIST‐T1‐*Scrambled* cells (Figure 2C). Overall, these results suggest that LIX1 promotes GIST cell proliferation and migration in vitro.

In many GIST specimens (~85%), *KIT* harbours gain‐of‐function mutations that cause ligand‐independent auto‐activation of the receptor.^30^, ^31^ Constitutive activation of the KIT receptor and of the downstream signalling pathways promotes tumour cell proliferation and uncontrolled growth.^32^, ^33^, ^34^ Therefore, we assessed KIT phosphorylation, a measure of KIT signalling activity. *LIX1* silencing decreased KIT phosphorylation (TYR703) level compared with GIST‐T1‐*Scrambled* cells, and also KIT expression, a signature of GIST cell identity (Figure 2D), although *KIT* transcript level was not changed (Figure S1).

We next investigated the mechanisms by which LIX1 regulates KIT. We previously reported that LIX1 regulates mesenchymal progenitor proliferation and differentiation through the Hippo mediator YAP1,^13^ which is also involved in the control of GIST cell proliferation.^35^ Therefore, we investigated the effect of *LIX1* silencing on the two Hippo effectors YAP1 and TAZ. LIX1 down‐regulation resulted in a marked decrease of YAP1/TAZ expression (Figure 2D) and function, as indicated by the lower levels of *CTGF* and *CYR61*, their transcriptional targets (Figure 2E). Furthermore, reducing YAP1 or TAZ expression (by *SiRNA*) (Figure 2F) and activity (using verteporfin, an inhibitor of YAP1‐TEAD/TAZ‐TEAD interactions) (Figure 2F) led to a marked decrease of KIT expression.

### 3.3 LIX1 down‐regulation induces a SMC‐specific transcriptional programme in GIST cells

**FIGURE 3:**
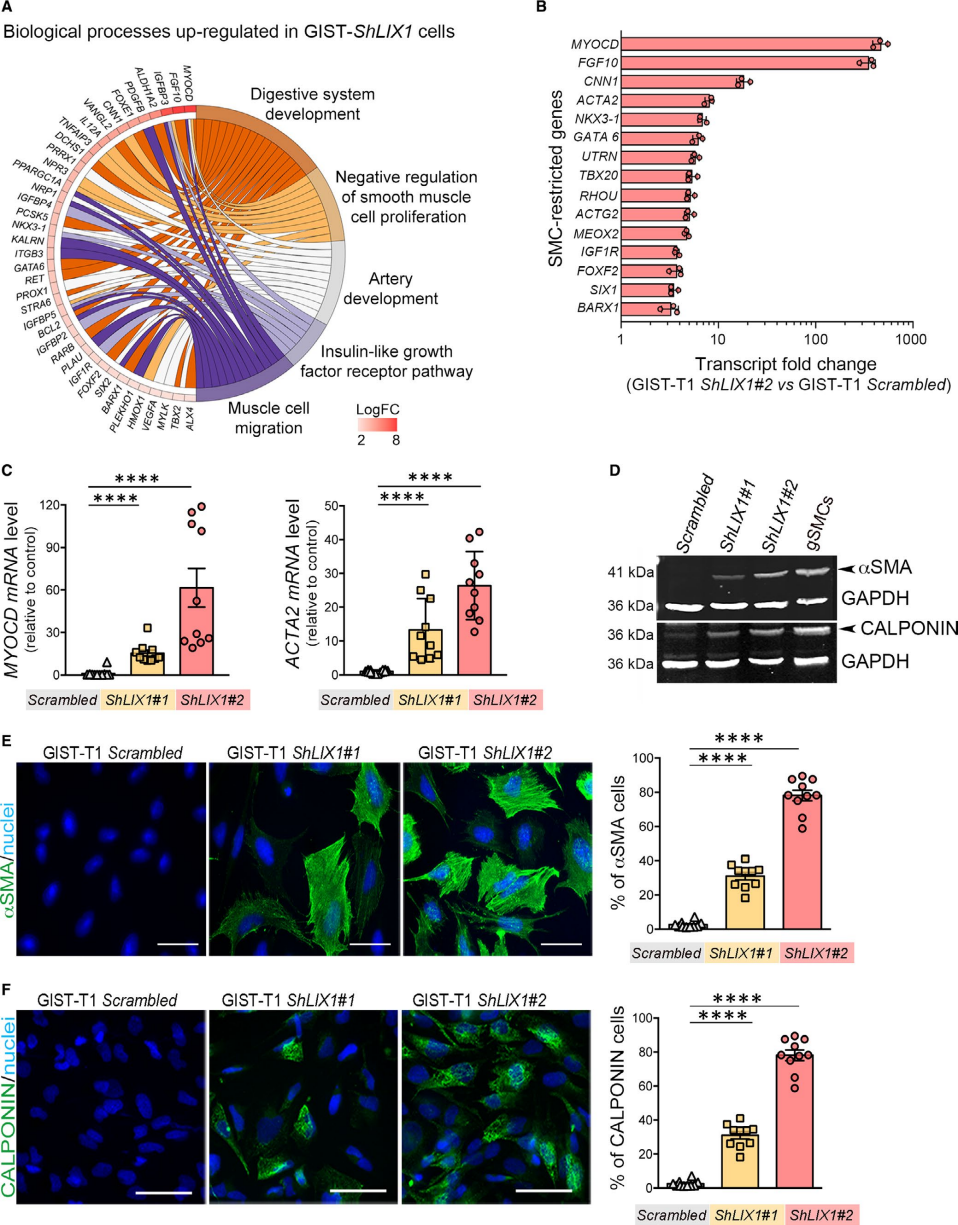
LIX1 down‐regulation reprogrammes KIT‐positive GIST cells to the SMC lineage. A, Transcriptional profiling of GIST‐T1‐*Scrambled* and GIST‐T1‐*ShLIX1* cells. Gene ontology enrichment analysis of up‐regulated genes and biological processes common to GIST‐T1‐*ShLIX1#1* and ‐*ShLIX1#2* in smooth muscle development. Data were from n = 3 GIST‐T1‐*Scrambled*, n = 3 GIST‐T1‐*ShLIX1#1* and n = 3 ‐*ShLIX1#2* (n = 3) independent samples. B, Transcript fold change of SMC‐restricted genes in GIST‐T1‐*ShLIX1* vs GIST‐T1‐*Scrambled* cells. The SMC gene list is based on previously published work (Table S5); *P* < .001. C, RT‐qPCR analysis of *MYOCD* and *ACTA2* relative mRNA expression in GIST‐T1‐*Scrambled* cells vs GIST‐T1‐*ShLIX1#1* and ‐*ShLIX1#2* cells. Data were normalized to the mean *GAPDH* and *RPLPO* transcript levels, and converted to fold changes. Graph represents the quantification of data of three independent experiments. Values are the mean ± SEM of GIST‐T1‐*Scrambled* cells (n = 14), GIST‐T1‐*ShLIX1#1* (n = 10) and GIST‐T1‐*ShLIX1#2* (n = 10) cells. ****P < .0001 (two‐tailed Mann–Whitney test). D, Representative western blot showing αSMA and CALPONIN levels in GIST‐T1‐*Scrambled*, GIST‐T1‐*ShLIX1#1* and ‐*ShLIX1#2* cells. Human gastric Smooth Muscle Cells (gSMCs) were used as positive control. Equal loading was verified by GAPDH expression. E, Immunofluorescence analysis of GIST‐T1‐*Scrambled*, GIST‐T1‐*ShLIX1#1* and ‐*ShLIX1#2* cells using anti‐αSMA antibodies. Nuclei were visualized with Hoechst. Scale bar, 50 μm. Quantification of the number of αSMA‐positive cells in GIST‐T1‐*Scrambled*, GIST‐T1‐*ShLIX1#1* and ‐*ShLIX1#2* cells. Values are the mean ± SEM of GIST‐T1‐*Scrambled* (n = 701), GIST‐T1‐*ShLIX1#1* (n = 494) and GIST‐T1‐*ShLIX1#2* (n = 186) cells. ****P < .0001 (two‐tailed Mann–Whitney test). F, Immunofluorescence analysis of GIST‐T1‐*Scrambled*, GIST‐T1‐*ShLIX1#1* and ‐*ShLIX1#2* cells using anti‐CALPONIN antibodies. Nuclei were visualized with Hoechst. Scale bars, 50 μm. Quantifications of the number of CALPONIN‐positive GIST‐T1‐*Scrambled*, GIST‐T1‐*ShLIX1#1* and ‐*ShLIX1#2* cells. Values are the mean ± SEM of GIST‐T1‐*Scrambled* (n = 467), GIST‐T1‐*ShLIX1#1* (n = 419) and ‐*ShLIX1#2* (n = 430) cells. ****P < .0001 (two‐tailed Mann–Whitney test)

As *LIX1* silencing was associated with loss of KIT expression, we wanted to thoroughly describe GIST‐T1‐*ShLIX1* cell identity. To this aim, we determined the gene expression profile of GIST‐T1 cells in function of LIX1 expression (GIST‐T1‐*ShLIX1#1*, GIST‐T1‐*ShLIX1#2* and vs GIST‐T1‐*Scrambled* cells) using a high‐throughput RNA‐sequencing approach. Transcriptomic analyses revealed the differential expression of several GIST‐restricted genes (*CD34*, *ENG*) and genes involved in GIST aggressiveness (eg *ETV4*, *SLITRK3*) (Figure S2A,B; Tables S5 and S6). Unexpectedly, we observed the up‐regulation of *MYOCD*, a master regulator of SMC‐restricted gene expression,^36^ and of SMC‐restricted contractile genes in GIST‐T1‐*ShLIX1* cells (Figure 3A,B; Tables S7 and S8). We confirmed the induction of a SMC‐restricted‐transcriptional programme in GIST‐T1‐*ShLIX1* cells by RT‐qPCR analysis of *MYOCD* and *ACTA2* (which encodes alpha Smooth Muscle Actin (αSMA)). Moreover, immunoblot and immunofluorescence analyses confirmed the induction of contractile proteins, such as αSMA and CALPONIN (a protein involved in smooth muscle contractility), in GIST‐T1‐*ShLIX1* cells compared with GIST‐T1‐*Scrambled* cells (Figure 3D‐F). We observed the induction of SMC‐restricted genes also in GIST‐T1 cells in which *LIX1* was transiently down‐regulated (Figure S3A,B). As GISTs originate from KIT‐positive ICCs that share common mesenchymal progenitors with SMCs, these findings indicate that *LIX1* silencing promotes a phenotypic modulation of KIT‐positive GIST cells towards the SMC lineage.

### 3.4 LIX1 silencing reduces GIST aggressiveness in vivo

**FIGURE 4:**
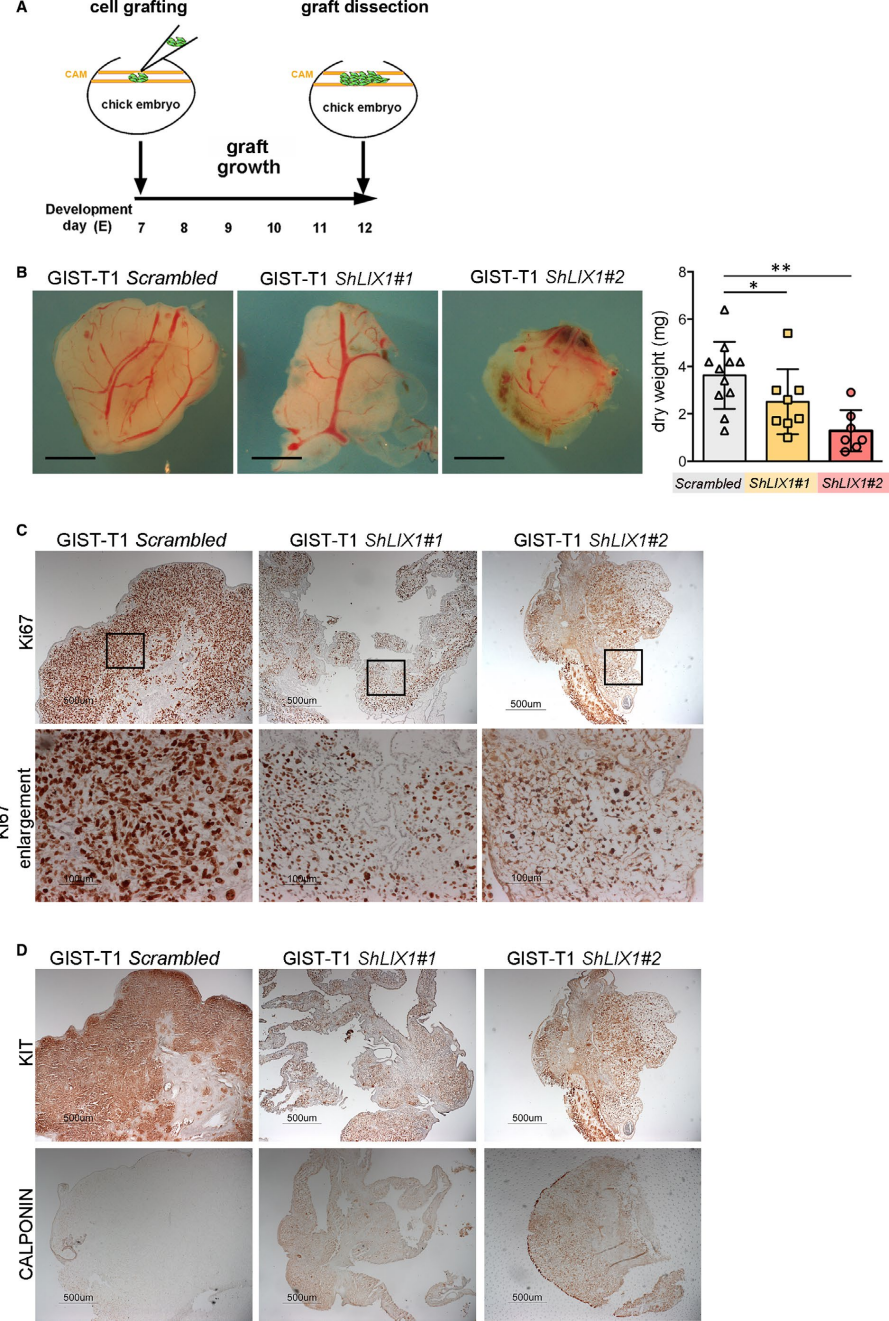
*LIX1* silencing reduces GIST aggressive phenotype in vivo. A, Schematic representation of the approach. GIST‐T1‐*Scrambled*, GIST‐T1‐*ShLIX1#1* or ‐*ShLIX1#2* cells were grafted in the ChorioAllantoic Membrane (CAM) of E7 chicken embryos and grafts were allowed to grow for 5 d when individual grafts were removed and analysed. B, Representative images (left panels) and dry weight (right panel) of GIST‐T1‐*Scrambled* and GIST‐T1‐*ShLIX1* cell grafts at E12. Scale bars, 2 mm. Values are the mean ± SEM of GIST‐T1‐*Scrambled* (n = 11), GIST‐T1‐*ShLIX1#1* (n = 8) and GIST‐T1‐*ShLIX1#2* cell grafts (n = 7). *P < .05 and **P < .01 (one‐tailed Mann–Whitney test). C, KI‐67 immunostaining of GIST‐T1‐*Scrambled* and GIST‐T1‐*ShLIX1* graft sections. Scale bars, 500 μm. Enlargement, 100 μm. D, KIT and CALPONIN expression in GIST‐T1‐*Scrambled* and GIST‐T1‐*ShLIX1* grafts. Scale bars, 500 μm

Our previous results demonstrated that in response to *LIX1* silencing, GIST cells lose KIT expression, a signature of GIST cell identity, and acquire smooth muscle features, a quality associated with reduced proliferative and invasive capacities. Therefore, we assessed the effect of *LIX1* silencing in vivo using the CAM assay, an established in vivo tumour model.^37^ After graft of GIST‐T1‐*Scrambled*, GIST‐T1‐*ShLIX1#1* and GIST‐T1‐*ShLIX1#2* cells in the CAM of E7 chicken embryos, we allowed tumour growth until E12 (Figure 4A) and then dissected and analysed them (Figure 4B). The graft dry weight was lower in GIST‐T1‐*ShLIX1* than GIST‐T1‐*Scrambled* cell grafts (Figure 4B). Moreover, immunohistochemical analysis of the tumour tissue showed that *LIX1* silencing significantly decreased cell proliferation (KI67) (Figure 4C and Figure S4). Conversely, CALPONIN expression was significantly increased and KIT expression reduced in GIST‐T1‐*ShLIX1* compared with GIST‐T1‐*Scrambled* cell grafts (Figure 4D). Collectively, our data indicate that *LIX1* silencing promotes a phenotypic modulation of KIT‐positive GIST cells towards the SMC lineage and subsequently reduces GIST malignant phenotype.

## 4 DISCUSSION

Gastrointestinal stromal tumours result from the deregulated proliferation of transformed KIT‐positive cells that originate from mesenchymal progenitors. We previously identified the *LIX1* gene as a marker of the immature state of stomach muscle cells and demonstrated that LIX1 controls mesenchymal progenitor proliferation and differentiation upstream of YAP1.^13^ As neoplastic transformation requires changes in cell identity and acquisition of progenitor‐like features,^28^ here we assessed *LIX1* expression in GISTs. Our findings highlight LIX1 fundamental role in GIST pathophysiology, as a rheostat of GIST cell identity by controlling YAP1/TAZ levels and subsequently regulating the signalling cascades that modulate fate decisions within the SMC lineage.

**FIGURE 5:**
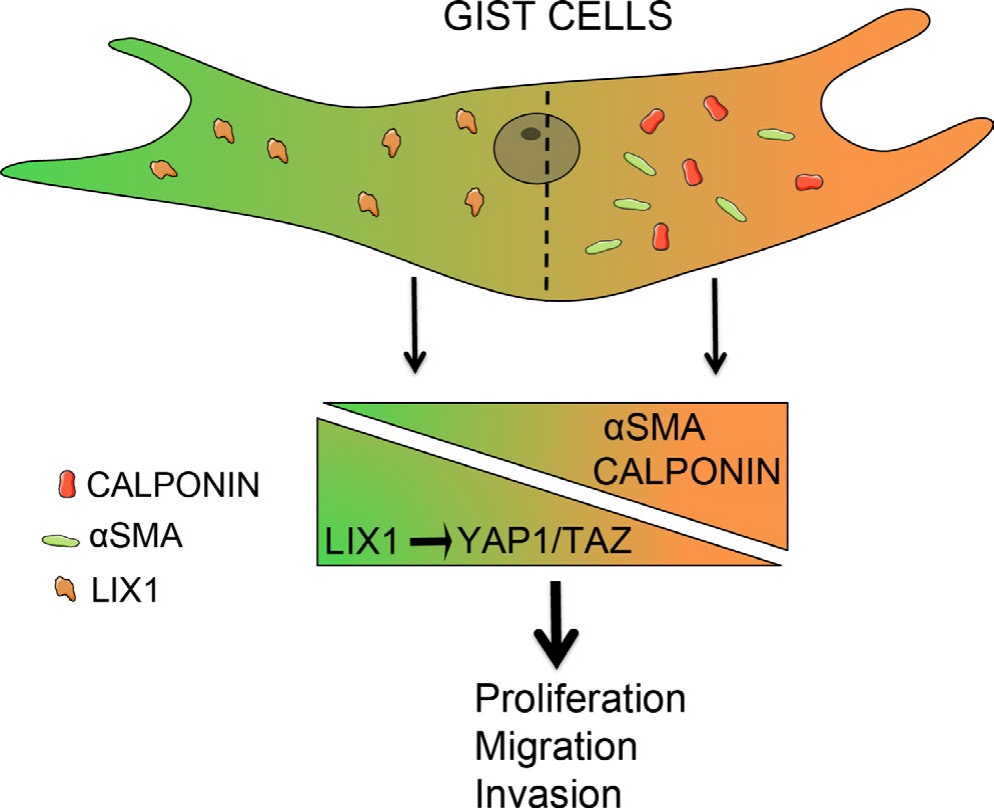
Model illustrating how LIX1 controls GIST malignant phenotype

We show that LIX1 is strongly expressed in GISTs and that its expression is associated with poor prognosis. We also demonstrated that LIX1 regulates YAP1/TAZ levels and activity, and controls the proliferative and invasive capacities of GIST cells. *Lowfat*, the arthropod homologue of *LIX1*, was characterized as a component of the Hippo pathway through its interaction with the atypical cadherins *fat* and *dachsous*.^38^, ^39^ Over the past two decades, the Hippo pathway has been connected with developmental processes and with tissue repair that are intimately linked to the function of tissue‐specific progenitor cells. Many studies have shown that the Hippo pathway controls cell proliferation by negatively regulating its downstream effectors YAP1 and TAZ.^40^ Constitutive activation of YAP1/TAZ has been observed in many human tumours.^41^ We found that reducing YAP1/TAZ protein level or activity results in a marked decrease of KIT expression. Although YAP1 regulates the proliferation of GIST cells,^35^ to our knowledge, our study is the first to reveal a control of YAP1/TAZ in KIT‐mediated GIST development. We found that *LIX1* silencing results in a marked decrease of YAP1/TAZ levels and function, and demonstrated that reducing YAP1 or TAZ expression or activity leads to a marked decrease of KIT expression. It is tempting to hypothesize that LIX1 regulates KIT protein level upstream of YAP1/TAZ (Figure 5). Additional studies are now required to precisely determine how LIX1 regulates YAP1/TAZ levels. The human *LIX1* gene encodes a 282‐amino acid protein that includes a double‐stranded RNA‐binding domain. This suggests that LIX1 could be involved in mRNA or micro‐RNA processing.^39^ Accordingly, miR‐506 and miR‐375 regulate YAP1 expression.^42^, ^43^ We found that in response to *LIX1* silencing, GIST cells lose KIT expression and subsequently acquire features of differentiated SMCs, a quality associated with reduced proliferative and invasive capacities. These findings provide the proof‐of‐concept that differentiation therapy could be a potential treatment strategy for GISTs.

In summary, our data demonstrate that *LIX1* silencing in GIST cells results in their reprogramming to SMCs, thereby limiting their aggressive potential. These findings highlight LIX1 potential as a drug target in GISTs in the framework of a differentiation therapy strategy for this aggressive digestive mesenchymal malignancy.

## CONFLICT OF INTEREST

The authors disclose no potential conflicts of interest.

## AUTHOR CONTRIBUTION

**Amandine Guérin:** Formal analysis (equal); Investigation (equal). **Delphine Martire:** Formal analysis (equal); Investigation (equal). **Eva Trenquier:** Formal analysis (equal); Methodology (equal). **Tom Lesluyes:** Formal analysis (equal); Methodology (equal). **Sébastien Sagnol:** Conceptualization (equal); Formal analysis (equal). **Marine Pratlong:** Conceptualization (equal); Investigation (equal). **Elise Lefebvre:** Formal analysis (equal). **Fréderic Chibon:** Methodology (equal); Resources (equal). **Pascal de Santa Barbara:** Funding acquisition (equal); Project administration (equal); Writing‐original draft (equal); Writing‐review & editing (equal). **Sandrine Faure:** Conceptualization (equal); Funding acquisition (equal); Methodology (equal); Project administration (equal); Supervision (equal); Writing‐original draft (equal); Writing‐review & editing (equal).

## Supporting information

Supplementary Material

## Data Availability

The data that support the findings of this study are available from the corresponding author upon request.
