# Supplementary material for: LIX1 regulates YAP activity and controls gastrointestinal cancer cell plasticity
Source: J Cell Mol Med. 2020 Jul 7;24(16):9244–54. doi: 10.1111/jcmm.15569 (PMC7417687; doi:10.1111/jcmm.15569)
Supplement: Supplementary file 1 — Supplementary Material [file JCMM-24-9244-s001.pdf]

Legends to Supplementary Figures

Supplementary Fig 1. LIX1 downregulation does not affect *KIT* transcript levels.

RT-qPCR analysis of *KIT* relative mRNA expression. Data were normalized to the mean *GAPDH* and *RPLPO* expression, and converted to fold changes. Values are the mean ± SEM of *n* =19 GIST-T1-*Scrambled*, *n=10* GIST-T1-*ShLIX1#1* and *ShLIX1#2* cells. *ns*, not significant by two-tailed Mann–Whitney tests.

Supplementary Fig 2. Transcriptional profiling of GIST-T1-*Scrambled* and GIST-T1-*ShLIX1* cells. **(A)** Gene ontology enrichment analysis of downregulated genes and biological processes common to GIST-T1-*ShLIX1#1* and -*ShLIX1#2* cells. Data are from *n*=3 GIST-T1-*Scrambled*, *n*=3 GIST-T1-*ShLIX1#1* and *n*=3-*ShLIX1#2* independent samples. **(B)** Transcript fold change of genes restricted to GIST cells or involved in their agressiveness in GIST-T1-*ShLIX1* versus GIST-T1-*Scrambled* cells (P< 0.001). The GIST gene list is based on previously published work (Supplementary Table 4).

Supplementary Fig 3. Transient *LIX1* silencing promotes phenotypic modulation of GIST cells toward the SMC lineage. GIST-T1 cells were analysed at 72 hours post-transfection with the *Scrambled* control sequence (GIST-T1-*Scrambled*) or *ShLIX1#2* (GIST-T1-*ShLIX1#2*). **(A)** Immunofluorescence analysis of GFP-positive cells using anti-αSMA antibodies. Nuclei were visualized with Hoechst. Scale bar, 50μm. Graph represents the quantification of data from three independent experiments. Values are the mean ± SEM of *n=500* GIST-T1-*Scrambled* cells and *n=199* for GIST-T1-*ShLIX1#2* cells. ****P <0.0001 (two-tailed Mann–Whitney test). **(B)** Representative Western blot using anti-αSMA, KIT and phosphorylated KIT (pKIT) antibodies. Equal loading was verified by GAPDH expression.

Supplementary Fig 4. ***LIX1*** **silencing reduces GIST malignant phenotype** ***in vivo.*** Haematoxylin-eosin staining of GIST-T1-*Scrambled* and GIST-T1-*ShLIX1* grafts. 500µm. Enlargement, 100µm.

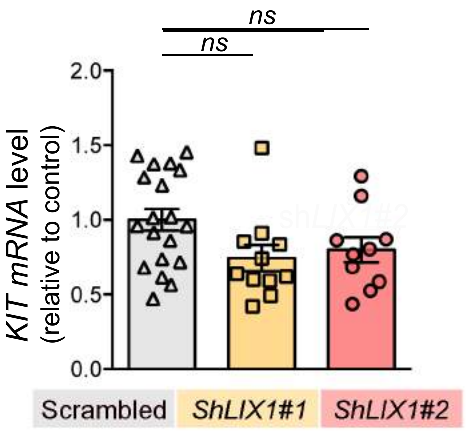

Supplementary Figure 1

A

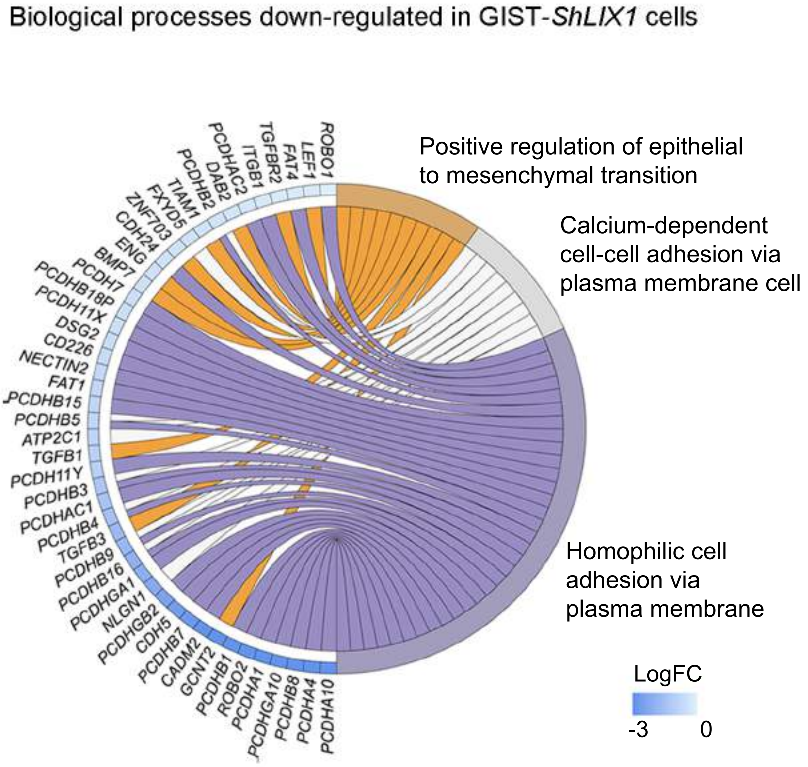

B

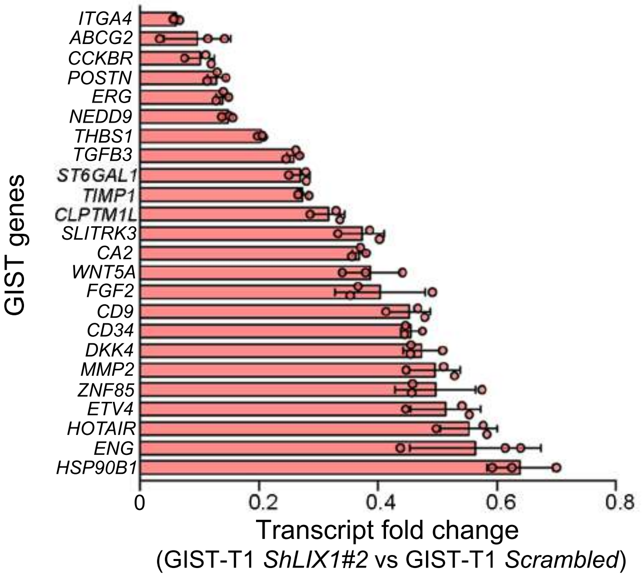

Supplementary Figure 2

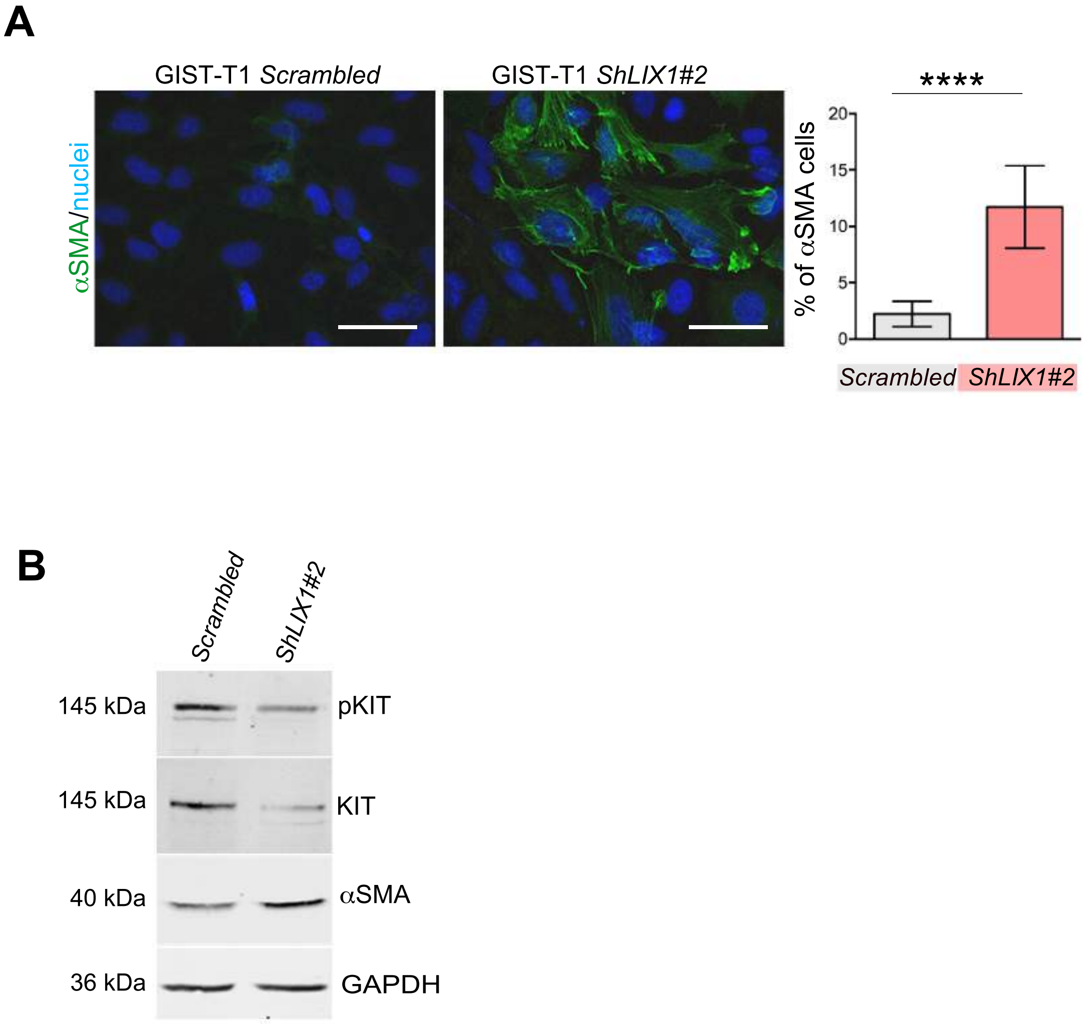

Supplementary Figure 3

A

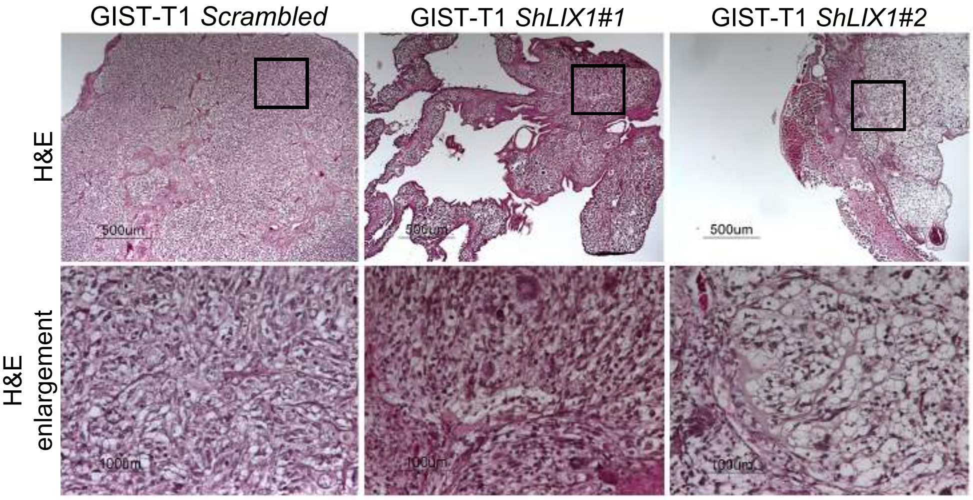

Supplementary Figure 4

Supplementary Table 1 : TMA Human Gastrointestinal Tumor (DAA2)

Age	Sex	Organ	Pathology	Grade
45	m	Jejunum	GIST	high
57	f	Jejunum	GIST	high
77	f	Stomach	GIST	high
60	f	Stomach	GIST	high
59	m	Jejunum	GIST	high
58	m	Small Intestine	GIST	high
65	f	Stomach	GIST	low
61	m	Stomach	GIST	high
65	f	Stomach	GIST	high
44	m	Small Intestine	GIST	high
50	f	Duodenum	GIST	intermediate
50	m	Stomach	GIST	low
68	m	Stomach	GIST	high
34	m	Duodenum	GIST	high
61	m	Stomach	GIST	intermediate
58	f	Stomach	GIST	high
57	f	Stomach	GIST	low
40	m	Stomach	GIST	high
66	f	Rectum	GIST	high
45	f	Abdominal cavity	GIST	high
66	m	Stomach	GIST	high
83	f	Stomach	GIST	high
60	f	Jejunum	GIST	intermediate
65	m	Jejunum	GIST	high
56	f	Duodenum	GIST	low
85	f	Stomach	GIST	high
67	f	Duodenum	GIST	intermediate
52	m	Stomach	GIST	high
65	m	Stomach	GIST	low
65	m	Ileum	GIST	intermediate
45	f	Stomach	GIST	low
55	m	Rectum	GIST	high
71	m	Stomach	GIST	high
67	f	Stomach	GIST	intermediate
62	f	Stomach	GIST	intermediate
48	m	Stomach	GIST	high
80	m	Small Intestine	GIST	low
60	m	Small Intestine	GIST	low
43	m	Duodenum	GIST	low
53	f	Stomach	GIST	high

Supplementary Table 2: TMA Human Gastrointestinal Tumor (A225)

Age	Sex	Organ	Pathology	Grade
61	m	Stomach	GIST	high
67	m	Jejunum	GIST	high
62	m	Stomach	GIST	high
44	m	Stomach	GIST	intermediate
46	f	Stomach	GIST	intermediate
62	m	Stomach	GIST	high
60	m	Stomach	GIST	high
68	f	Stomach	GIST	high
35	f	Stomach	GIST	intermediate
50	m	Stomach	GIST	intermediate
56	m	Stomach	GIST	high
64	m	Stomach	GIST	high
26	f	Stomach	GIST	high
51	f	Stomach	GIST	intermediate
43	m	Stomach	GIST	intermediate
49	m	Stomach	GIST	high
73	m	Stomach	GIST	high
75	m	Stomach	GIST	high
45	m	small Intestine	GIST	intermediate
34	m	small Intestine	GIST	high
80	f	small Intestine	GIST	high
49	f	small Intestine	GIST	intermediate
61	f	small Intestine	GIST	intermediate
60	m	small Intestine	GIST	high
41	f	small Intestine	GIST	high
40	f	small Intestine	GIST	high
53	f	small Intestine	GIST	intermediate
50	m	duodenum	GIST	intermediate
59	m	colon	GIST	high
63	f	stomach	GIST	high
71	m	Esophagus	GIST	very low
57	f	stomach	GIST	low
40	m	small Intestine	GIST	low
68	f	stomach	GIST	very low
64	f	stomach	GIST	very low
59	m	duodenum	GIST	low
27	m	duodenum	GIST	low

Supplementary Table 3 : Human gene-specific primers used for qRT-PCR.

Targets	Forward primer (5'-3')	Reverse primer (5'-3')	Amplicon (bp)
ACTA2	TTC AAT GTC CCA GCC ATG TA	GAA GGA ATA GCC ACG CTC AG	222
CTGF	AGG AGT GGG TGT GTG AC	CCA GGC AGT TGG CTC TAA	117
CYR61	GGA GCC TCG CAT CCT AT	ATT GGT AAC TCG TGT GGA GA	115
GAPDH	CCA TCT TCC AGG AGC GAG	CTT GAG GCT GTT GTC ATA CT	212
KIT	CCT TTG CTG ATT GGT TTC G	AGG AAG TTG TGT TGG GTC TA	162
LIX1	GTG TTG GGG CCT ATC ACT AC	GAG ATG ACT TCC TGT CGG G	151
MYOCD	ATC AAT GAA CTC ACC TGG AAA [illegible]	GCG GCT TCT TCT CTG AAC A	106
RPLPO	TCA TCC AGC AGG TGT TCG	AGC AAG TGG GAA GGT GTA A	224

Supplementary Table 4 : List of Antibodies.

-Polyclonal rabbit LIX1 antibody (Aviva Systems Biology Cat# ARP53147_P050, RRID:AB_2045922) : WB or IF

-Monoclonal mouse CALPONIN antibody (Santa Cruz Biotechnology Cat# sc-58707, RRID:AB_781770)

-Monoclonal rabbit KIT antibody (DB Biotech Cat# DB 062, RRID:AB_2315699)

-Monoclonal rabbit Phospho-KIT (Tyr703) antibody (Cell Signaling Technology Cat# 3073, RRID:AB_1147635)

-Monoclonal mouse αSMA antibody (Abcam Cat# ab7817, RRID:AB_262054)

Supplementary Table 5 : Fold changes and *p* values of down-regulated GIST-genes in GIST-T1-*ShLIX1#2* cells

gene	fold change	P value
ITGA4	0,060400405	0
ABCG2	0,096456789	4,67E-12
CCKBR	0,101814296	3,10E-64
POSTN	0,129035341	1,48E-185
ERG	0,139089178	5,96E-35
NEDD9	0,147854231	1,16E-157
THBS1	0,203574819	1,03E-236
TGFB3	0,258737928	3,55E-87
ST6GAL1	0,26943929	3,39E-68
TIMP1	0,272738166	3,04E-107
CLPTM1L	0,317138093	1,98E-81
SLITRK3	0,373849993	1,67E-34
CA2	0,368884805	1,01E-94
WNT5A	0,386897772	3,98E-35
FGF2	0,403766753	4,53E-25
CD9	0,455298049	1,60E-32
CD34	0,453028571	7,84E-40
DKK4	0,473075933	1,04E-35
MMP2	0,49554905	6,55E-28
ZNF85	0,496661553	4,24E-16
ETV4	0,513723362	1,02E-15
HOTAIR	0,552654429	1,25E-07
ENG	0,563888458	1,15E-08
HSP90B1	0,639033195	2,69E-11

Supplementary Table 6 : List of selected genes expressed in GISTs

ABCG2

Koo DH, Ryu MH, Ryoo BY, Beck MY, Na YS, Shin JG, Lee SS, Kim EY, Kang YK. Association of ABCG2 polymorphism with clinical efficacy of imatinib in patients with gastrointestinal stromal tumor. Cancer Chemother Pharmacol. 2015 Jan;75(1):173-82. doi: 10.1007/s00280-014-2630-6.

CA2

Parkkila S, Lasota J, Fletcher JA, Ou WB, Kivelä AJ, Nuorva K, Parkkila AK, Ollikainen J, Sly WS, Waheed A, Pastorekova S, Pastorek J, Isola J, Miettinen M. Carbonic anhydrase II. A novel biomarker for gastrointestinal stromal tumors. Mod Pathol. 2010 May;23(5):743-50. doi: 10.1038/modpathol.2009.189.

CCKBR

Quattrone A, Dewaele B, Wozniak A, Bauters M, Vanspauwen V, Floris G, Schöffski P, Chibon F, Coindre JM, Sciot R, Debiec-Rychter M. Promoting role of cholecystokinin 2 receptor (CCK2R) in gastrointestinal stromal tumour pathogenesis. J Pathol. 2012 Dec;228(4):565-74. doi: 10.1002/path.4071.

CD9

Setoguchi T, Kikuchi H, Yamamoto M, Baba M, Ohta M, Kamiya K, Tanaka T, Baba S, Goto-Inoue N, Setou M, Sasaki T, Mori H, Sugimura H, Konno H. Microarray analysis identifies versican and CD9 as potent prognostic markers in gastric gastrointestinal stromal tumors. Cancer Sci. 2011 Apr;102(4):883-9. doi: 10.1111/j.1349-7006.2011.01872.x.

CD34

Miettinen M, Virolainen M, Maarit-Sarlomo-Rikala. Gastrointestinal stromal tumors--value of CD34 antigen in their identification and separation from true leiomyomas and schwannomas. Am J Surg Pathol. 1995 Feb;19(2):207-16.

CLPTM1L

Zhang R, Zhao J, Xu J, Liu F, Xu Y, Bu X, Dai C, Song C. Genetic variations in the TERT and CLPTM1L gene region and gastrointestinal stromal tumors risk. Oncotarget. 2015 Oct 13;6(31):31360-7. doi: 10.18632/oncotarget.5153.

DKK4

Zeng S, Seifert AM, Zhang JQ, Cavnar MJ, Kim TS, Balachandran VP, Santamaria-Barria JA, Cohen NA, Beckman MJ, Medina BD, Rossi F, Crawley MH, Loo JK, Maltbaek JH, Besmer P, Antonescu CR, DeMatteo RP. Wnt/β-catenin Signaling Contributes to Tumor Malignancy and Is Targetable in Gastrointestinal Stromal Tumor. Mol Cancer Ther. 2017 Sep;16(9):1954-1966. doi: 10.1158/1535-7163.MCT-17-0139.

ENG

Gromova P, Rubin BP, Thys A, Cullus P, Erneux C, Vanderwinden JM. ENDOGLIN/CD105 is expressed in KIT positive cells in the gut and in gastrointestinal stromal tumours.J Cell Mol Med. 2012 Feb;16(2):306-17. doi: 10.1111/j.1582-4934.2011.01315.

ERG
Nannini M, Astolfi A, Urbini M, Indio V, Santini D, Heinrich MC, Corless CL, Ceccarelli C, Saponara M, Mandrioli A, Lolli C, Ercolani G, Brandi G, Biasco G, Pantaleo MA. Integrated genomic study of quadruple-WT GIST (KIT/PDGFRA/SDH/RAS pathway wild-type GIST). BMC Cancer. 2014 Sep 20;14:685. doi: 10.1186/1471-2407-14-685.

ETV4
Zeng S, Seifert AM, Zhang JQ, Kim TS, Bowler TG, Cavnar MJ, Medina BD, Vitiello GA, Rossi F, Loo JK, Param NJ, DeMatteo RP. ETV4 collaborates with Wnt/β-catenin signaling to alter cell cycle activity and promote tumor aggressiveness in gastrointestinal stromal tumor. Oncotarget. 2017 Dec 11;8(69):114195-114209. doi: 10.18632/oncotarget.23173.

FGF2
-Li F, Huynh H, Li X, Ruddy DA, Wang Y, Ong R, Chow P, Qiu S, Tam A, Rakiec DP, Schlegel R, Monahan JE, Huang A. FGFR-Mediated Reactivation of MAPK Signaling Attenuates Antitumor Effects of Imatinib in Gastrointestinal Stromal Tumors. Cancer Discov. 2015 Apr;5(4):438-51. doi: 10.1158/2159-8290.CD-14-0763.
-Javidi-Sharifi N, Traer E, Martinez J, Gupta A, Taguchi T, Dunlap J, Heinrich MC, Corless CL, Rubin BP, Druker BJ, Tyner JW. Crosstalk between KIT and FGFR3 Promotes Gastrointestinal Stromal Tumor Cell Growth and Drug Resistance. Cancer Res. 2015 Mar 1;75(5):880-91. doi: 10.1158/0008-5472.CAN-14-0573.

HOTAIR
Niinuma T, Suzuki H, Nojima M, Nosho K, Yamamoto H, Takamaru H, Yamamoto E, Maruyama R, Nobuoka T, Miyazaki Y, Nishida T, Bamba T, Kanda T, Ajioka Y, Taguchi T, Okahara S, Takahashi H, Nishida Y, Hosokawa M, Hasegawa T, Tokino T, Hirata K, Imai K, Toyota M, Shinomura Y. Upregulation of miR-196a and HOTAIR drive malignant character in gastrointestinal stromal tumors. Cancer Res. 2012 Mar 1;72(5):1126-36. doi: 10.1158/0008-5472.CAN-11-1803.

HSP90B1
Nakatani H, Kobayashi M, Jin T, Taguchi T, Sugimoto T, Nakano T, Hamada S, Araki K. STI571 (Glivec) inhibits the interaction between c-KIT and heat shock protein 90 of the gastrointestinal stromal tumor cell line, GIST-T1. Cancer Sci. 2005 Feb;96(2):116-9.

ITGA4
Pulkka OP, Mpindi JP, Tynninen O, Nilsson B, Kallioniemi O, Sihto H, Joensuu H. Clinical relevance of integrin alpha 4 in gastrointestinal stromal tumours. J Cell Mol Med. 2018 Apr;22(4):2220-2230. doi: 10.1111/jcmm.13502.

MMP2
Long ZW, Wu JH, Cai-Hong, Wang YN, Zhou Y. MiR-374b Promotes Proliferation and Inhibits Apoptosis of Human GIST Cells by Inhibiting PTEN through Activation of the PI3K/Akt Pathway. Mol Cells. 2018 Jun;41(6):532-544. doi: 10.14348/molcells.2018.2211.

NEDD9
Rink L, Ochs MF, Zhou Y, von Mehren M, Godwin AK. ZNF-mediated resistance to imatinib mesylate in gastrointestinal stromal tumor. PLoS One. 2013;8(1):e54477. doi: 10.1371/journal.pone.0054477.

POSTN, TGFB3
Rink L, Ochs MF, Zhou Y, von Mehren M, Godwin AK. ZNF-mediated resistance to imatinib mesylate in gastrointestinal stromal tumor. PLoS One. 2013;8(1):e54477. doi: 10.1371/journal.pone.0054477.

SLITRK3
Wang CJ, Zhang ZZ, Xu J, Wang M, Zhao WY, Tu L, Zhuang C, Liu Q, Shen YY, Cao H, Zhang ZG. SLITRK3 expression correlation to gastrointestinal stromal tumor risk rating and prognosis. World J Gastroenterol. 2015 Jul 21;21(27):8398-407. doi: 10.3748/wjg.v21.i27.8398.

ST6GAL1
Takahashi T, Naka T, Fujimoto M, Serada S, Horino J, Terabe F, Hirota S, Miyoshi E, Hirai T, Nakajima K, Nishitani A, Souma Y, Sawa Y, Nishida T. Aberrant expression of glycosylation in juvenile gastrointestinal stromal tumors. Proteomics Clin Appl. 2008 Sep;2(9):1246-54. doi: 10.1002/prca.200700119.

THBS1
Wang WJ, Li HT, Yu JP, Li YM, Han XP, Chen P, Yu WW, Chen WK, Jiao ZY, Liu HB. Identification of key genes and associated pathways in KIT/PDGFRA wild‑type gastrointestinal stromal tumors through bioinformatics analysis. Mol Med Rep. 2018 Nov;18(5):4499-4515. doi: 10.3892/mmr.2018.9457.

TIMP1
Liang YM, Li XH, Li WM, Lu YY. Prognostic significance of PTEN, Ki-67 and CD44s expression patterns in gastrointestinal stromal tumors. World J Gastroenterol. 2012 Apr 14;18(14):1664-71. doi: 10.3748/wjg.v18.i14.1664.

WNT5A
Ma MZ, Zhuang C, Yang XM, Zhang ZZ, Ma H, Zhang WM, You H, Qin W, Gu J, Yang S, Cao H, Zhang ZG. CTHRC1 acts as a prognostic factor and promotes invasiveness of gastrointestinal stromal tumors by activating Wnt/PCP-Rho signaling. Neoplasia. 2014 Mar;16(3):265-78, 278.e1-13. doi: 10.1016/j.neo.2014.03.001.

ZNF85
Strauss LG, Dimitrakopoulou-Strauss A, Koczan D, Pan L, Hohenberger P. Correlation of dynamic PET and gene array data in patients with gastrointestinal stromal tumors. ScientificWorldJournal. 2012;2012:721313. doi: 10.1100/2012/721313.

Supplementary Table 7 : Fold changes and *p* values of up-regulated SMC-genes in GIST-T1-ShLIX1#2

gene	fold change	P value
MYOCD	467,230754	1,84E-16
FGF10	349,745619	3,81E-11
CNN1	18,1643795	6,87E-18
ACTA2	8,06630005	1,14E-239
NKX3-1	6,82935245	1,13E-50
GATA6	6,2022628	1,88E-13
UTRN	5,7725972	7,44E-114
TBX20	5,34393998	6,07E-38
RHOU	5,12863604	1,18E-57
ACTG2	5,03061475	7,04E-62
MEOX2	4,71724598	9,96E-38
IGF1R	3,72869753	1,17E-11
FOXF2	3,7228552	2,51E-19
SIX1	3,46267923	3,89E-46
BARX1	3,2420341	2,05E-14

Supplementary Table 8 : List of selected SMC-Genes

ACTA2

Notarnicola C, Rouleau C, Le Guen L, Virsolvy A, Richard S, Faure S, De Santa Barbara P. The RNA-binding protein RBPMS2 regulates development of gastrointestinal smooth muscle. Gastroenterology. 2012 Sep;143(3):687-697.e9. doi: 10.1053/j.gastro.2012.05.047.

ACTG2

Lehtonen HJ, Sipponen T, Tojkander S, Karikoski R, Järvinen H, Laing NG, Lappalainen P, Aaltonen LA, Tuupanen S. Segregation of a missense variant in enteric smooth muscle actin γ-2 with autosomal dominant familial visceral myopathy. Gastroenterology. 2012 Dec;143(6):1482-1491.e3. doi: 10.1053/j.gastro.2012.08.045. Epub 2012 Sep 6.

BARX1

Jayewickreme CD, Shivdasani RA. Control of stomach smooth muscle development and intestinal rotation by transcription factor BARX1. Dev Biol. 2015 Sep 1;405(1):21-32. doi: 10.1016/j.ydbio.2015.05.024.

CNN1

McKey J, Martire D, de Santa Barbara P, Faure S. LIX1 regulates YAP1 activity and controls the proliferation and differentiation of stomach mesenchymal progenitors. BMC Biol. 2016 Apr 28;14:34. doi: 10.1186/s12915-016-0257-2.

FGF10

-Mailleux AA, Kelly R, Veltmaat JM, De Langhe SP, Zaffran S, Thiery JP, Bellusci S. Fgf10 expression identifies parabronchial smooth muscle cell progenitors and is required for their entry into the smooth muscle cell lineage. Development. 2005 May;132(9):2157-66.
-Le Guen L, Notarnicola C, de Santa Barbara P. Intermuscular tendons are essential for the development of vertebrate stomach. Development. 2009 Mar;136(5):791-801. doi: 10.1242/dev.029942.

FOXF2

Bolte C, Ren X, Tomley T, Ustiyan V, Pradhan A, Hoggatt A, Kalin TV, Herring BP, Kalinichenko VV. Forkhead box F2 regulation of platelet-derived growth factor and myocardin/serum response factor signaling is essential for intestinal development. J Biol Chem. 2015 Mar 20;290(12):7563-75. doi: 10.1074/jbc.M114.609487. Epub 2015 Jan 28.

GATA6

Zeng L, Carter AD, Childs SJ. miR-145 directs intestinal maturation in zebrafish. Proc Natl Acad Sci U S A. 2009 Oct 20;106(42):17793-8. doi: 10.1073/pnas.0903693106

IGF1R

Zeeh JM, Ennes HS, Hoffmann P, Procaccino F, Eysselein VE, Snape WJ Jr, McRoberts JA. Expression of insulin-like growth factor I receptors and binding proteins by colonic smooth muscle cells. Am J Physiol. 1997 Mar;272(3 Pt 1):G481-7.

MEOX2

Skopicki HA, Lyons GE, Schatteman G, Smith RC, Andrés V, Schirm S, Isner J, Walsh K. Embryonic expression of the Gax homeodomain protein in cardiac, smooth, and skeletal muscle. Circ Res. 1997 Apr;80(4):452-62.

MYOCD
Huang J, Wang T, Wright AC, Yang J, Zhou S, Li L, Yang J, Small A, Parmacek MS. Myocardin is required for maintenance of vascular and visceral smooth muscle homeostasis during postnatal development. Proc Natl Acad Sci U S A. 2015 Apr 7;112(14):4447-52. doi: 10.1073/pnas.1420363112.

NKX3-1
Sun Q, Taurin S, Sethakorn N, Long X, Imamura M, Wang DZ, Zimmer WE, Dulin NO, Miano JM. Myocardin-dependent activation of the CArG box-rich smooth muscle gamma-actin gene: preferential utilization of a single CArG element through functional association with the NKX3.1 homeodomain protein. J Biol Chem. 2009 Nov 20;284(47):32582-90. doi: 10.1074/jbc.M109.033910. Epub 2009 Sep 21.

RHOU
Notarnicola C, Le Guen L, Fort P, Faure S, de Santa Barbara P. Dynamic expression patterns of RhoV/Chp and RhoU/Wrch during chicken embryonic development. Dev Dyn. 2008 Apr;237(4):1165-71. doi: 10.1002/dvdy.21507.

TBX20
Iio A, Koide M, Hidaka K, Morisaki T. Expression pattern of novel chick T-box gene, Tbx20. Dev Genes Evol. 2001 Dec;211(11):559-62.

UTRN
Nguyen TM, Ellis JM, Love DR, Davies KE, Gatter KC, Dickson G, Morris GE. Localization of the DMDL gene-encoded dystrophin-related protein using a panel of nineteen monoclonal antibodies: presence at neuromuscular junctions, in the sarcolemma of dystrophic skeletal muscle, in vascular and other smooth muscles, and in proliferating brain cell lines. J Cell Biol. 1991 Dec;115(6):1695-700.

SIX1
-El-Hashash AH, Al Alam D, Turcatel G, Rogers O, Li X, Bellusci S, Warburton D. Six1 transcription factor is critical for coordination of epithelial, mesenchymal and vascular morphogenesis in the mammalian lung. Dev Biol. 2011 May 15;353(2):242-58. doi: 10.1016/j.ydbio.2011.02.031. Epub 2011 Mar 6. Erratum in: Dev Biol. 2014 Jul 15;391(2):252-3.
-Nie X, Sun J, Gordon RE, Cai CL, Xu PX. SIX1 acts synergistically with TBX18 in mediating ureteral smooth muscle formation. Development. 2010 Mar;137(5):755-65. doi: 10.1242/dev.045757
